# Perception Versus Actual Weight: Body Image Dissatisfaction as a Stronger Correlate of Anxiety and Depression than BMI Among Romanian Health Sciences Students

**DOI:** 10.3390/healthcare13233118

**Published:** 2025-12-01

**Authors:** Catalin Pleșea-Condratovici, Vlad Dionisie, Lavina-Alexandra Moroianu, Petrut-Stefan Serban, Victor Plesea-Condratovici, Manuela Arbune

**Affiliations:** 1 Morphological and Functional Sciences Department, Faculty of Medicine and Pharmacy, “Dunărea de Jos” University of Galaţi, 800008 Galati, Romania; catalin.plesea@ugal.ro; 2 Department of Psychiatry and Psychology, “Carol Davila” University of Medicine and Pharmacy, 020021 Bucharest, Romania; vlad.dionisie@umfcd.ro; 3 Pharmaceutical Sciences Department, Faculty of Medicine and Pharmacy, “Dunărea de Jos” University of Galaţi, 800201 Galati, Romania; 4 Faculty of Medicine and Pharmacy, “Dunărea de Jos” University of Galaţi, 800008 Galati, Romania; victormihaipc@gmail.com; 5 Clinical Medical Department, Faculty of Medicine and Pharmacy, “Dunărea de Jos” University of Galaţi, 800008 Galati, Romania; manuela.arbune@ugal.ro

**Keywords:** body image, body dissatisfaction, anxiety, depression, medical students, nursing students, Romania, EDE-Q, HADS

## Abstract

**Background**: The high prevalence of anxiety and depression among young adults constitutes a significant public health concern, with body image identified as a key psychological factor. However, the interplay between subjective metrics (perceived body image) and objective measures (Body Mass Index) remains insufficiently explored within specific populations, such as health sciences students in Eastern Europe. **Methods**: An observational, cross-sectional study was conducted on a sample of 137 medical and nursing students from a Romanian university. Validated instruments were employed: the Hospital Anxiety and Depression Scale (HADS) and the Eating Disorder Examination Questionnaire (EDE-Q). BMI was calculated from self-reported height and weight. Spearman’s correlation analyses and Mann–Whitney U tests were performed. **Results**: Subjective body image concerns, particularly those related to shape, weight, and eating, were found to be positively and significantly correlated with symptoms of anxiety and depression (all *p* < 0.05). In contrast, BMI exhibited no significant correlation with either of the HADS subscales. Although nursing students presented a higher mean BMI, no significant differences were recorded between the student groups with respect to psychological symptoms or body image concerns. **Conclusions**: Subjective dissatisfaction with body image is a more salient correlate of emotional distress than objective body mass in this cohort. Although the sample size was moderate, the observed associations were robust and statistically significant, underscoring the importance of subjective body image over objective metrics such as BMI in this academic population. These findings underscore the necessity for mental health interventions within university settings to focus on the perceptual and cognitive-affective aspects of body image, rather than exclusively on weight management.

## 1. Introduction

Anxiety and depression represent some of the most prevalent psychological disorders globally, with their incidence rising particularly among adolescents and young adults [1,2,3,4]. The most comprehensive umbrella review to date synthesised data from over 8.7 million university students worldwide and found pooled prevalence rates for mild depressive symptoms at 35.4% (95% CI: 33.9–36.9) and mild anxiety symptoms at 40.2% (95% CI: 37.4–43.1), with severe symptoms at 13.4% and 16.8%, respectively [5]. These emotional conditions arise from a complex interplay of biological predispositions, environmental stressors, and underlying psychological vulnerabilities [6,7,8]. Within this context, body image dissatisfaction has emerged as a significant factor in psychological well-being [9,10]. Body image is a multifaceted construct, encapsulating an individual’s perceptions, thoughts, and emotional responses to their physical appearance [10,11]. Meta-analytic reviews confirm a robust negative correlation between body satisfaction and symptoms of depression and anxiety among university students, establishing that body dissatisfaction is a significant predictor of these disorders [12].

A fundamental distinction must be drawn between objective anthropometric measures, such as the Body Mass Index (BMI), and the subjective psychological experience of one’s own body. Although BMI serves as a standardised metric for weight status, it does not adequately capture the complexity of body-related emotional and cognitive experiences [10,13]. There is a growing body of evidence to suggest that an individual’s subjective perceptions and feelings toward their body may serve as a more potent predictor of psychological distress than objective weight status alone [14,15].

The majority of research in this domain originates from Western countries, leaving a comparative deficit of data from Eastern Europe, a region undergoing unique socio-cultural transitions [16,17]. Studies from neighbouring countries provide an important regional context. Research in Poland indicates high body dissatisfaction and a pronounced desire to alter physical appearance, particularly among women [18,19]. In the Czech Republic and Slovakia, body dissatisfaction is also prevalent, especially in girls, and is not always correlated with actual obesity [20]. A study conducted in Moscow and Tiraspol revealed that the drivers of dissatisfaction differ by gender: males were dissatisfied with insufficient weight, whereas females were dissatisfied with excess weight [21]. This cultural friction could amplify the psychological distress associated with body image, rendering the subjective experience of the body particularly potent and relevant for investigation [22].

Within the Romanian context, research is limited but highly pertinent. A recent study demonstrated that Romanian medical students are at a higher risk of developing eating disorders compared to the general population, with familial pressure identified as a more powerful predictor than media pressure [23]. The specific population of medical and nursing students is particularly vulnerable, facing high levels of stress, academic pressure, and potential professional pressures related to health and physical appearance [24,25]. Other regional data indicate that a significant percentage of young people are at high risk for generalised anxiety disorder [26]. These findings suggest a significant mental health problem among young people in the region. A recent study combining institutional data with a national meta-analysis confirmed the high burden, finding that 34.0% of students at the same university presented with clinically significant depressive symptoms, with the pooled national prevalence standing at 19.99% [27].

This study, therefore, aims to address a significant gap in the literature by pursuing the following objectives:

1. To investigate the relationship between subjective body image concerns (measured with EDE-Q), objective body size (BMI), and symptoms of anxiety and depression (measured with HADS) in a sample of Romanian health sciences students.
2. To test the hypothesis that subjective body image concerns are more strongly associated with anxiety and depression than is BMI.
3. To explore potential differences in these variables between medical and nursing students.

While previous studies have focused primarily on Western populations, this research addresses a significant gap by providing robust data from an under-researched Eastern European academic context, using validated instruments and rigorous analytic methods. The moderate sample size is offset by the strong effect sizes and internal consistency observed.

## 2. Materials and Methods

### 2.1. Study Design

This study employed a cross-sectional, observational design to explore the associations between objective body size, subjective body image concerns, and psychological distress—specifically, symptoms of anxiety and depression—among university students. The study was conducted at a single university in Romania. The study followed the Strobe checklist. The checklist can be found in Supplementary Materials.

### 2.2. Participants and Study Procedure

A total of 137 students enrolled in the General Medicine and General Nursing undergraduate programmes at the Faculty of Medicine and Pharmacy, “Dunărea de Jos” University of Galați, participated in the study. Recruitment was conducted via internal university announcements and brief presentations during lectures. Data collection took place during the autumn semester of the 2024–2025 academic year. Inclusion criteria were: current enrolment in the faculty and age ≥ 18 years. No exclusion criteria were applied.

Data were collected via an anonymous online survey hosted on the PsyToolkit platform version: 3.6.2 [28,29]. Prior to participation, all respondents provided informed consent electronically. Participants were informed of the voluntary nature of the study, assured of the confidentiality and anonymity of their responses, and reminded of their right to withdraw at any time without penalty. The study was approved by the Ethics Committee of “Dunărea de Jos” University of Galați and was conducted in accordance with the ethical principles outlined in the Declaration of Helsinki.

The sample exhibited a significant female majority (77.4% women; 22.6% men), which reflects the general demographic trend in Romanian health sciences education. This imbalance is noted as a necessary limitation for the generalizability of the results to the broader population, particularly male students.

### 2.3. Measures

Symptoms of anxiety and depression were assessed using the Hospital Anxiety and Depression Scale (HADS) [30]. This 14-item self-report instrument is designed for use in general medical populations. The scale includes two subscales—anxiety (HADS-A) and depression (HADS-D)—each containing seven items scored on a 4-point Likert scale (0–3). Subscale scores range from 0 to 21, with higher scores indicating greater symptom severity. The HADS has demonstrated strong psychometric properties across diverse populations.

BMI was calculated based on self-reported height and weight using the standard formula (kg/m^2^). Subjective concerns about body image were measured using the Eating Disorder Examination Questionnaire (EDE-Q) [31]. This self-report instrument assesses attitudes and behaviours related to eating, shape, and weight over the past 28 days. Items are rated on a 7-point scale (0 = “No days” to 6 = “Every day”). The current study focused on the Shape Concern, Weight Concern, and Eating Concern subscales, with higher scores reflecting more pronounced body image distress. The EDE-Q is widely validated and has demonstrated reliability in both clinical and non-clinical samples.

### 2.4. Statistical Analysis

All data were analysed using IBM SPSS Statistics for Windows, version 27 [32]. The significance threshold was set at *p* < 0.05 for all tests. Descriptive statistics were calculated for demographic and psychometric variables. Spearman’s rank correlation coefficients were used to examine associations between subjective body image concerns, BMI, and psychological symptoms. Mann–Whitney U tests were applied to compare variables between student groups (Medicine vs. Nursing), given the non-normal distribution of most variables, as confirmed by the Shapiro–Wilk test.

## 3. Results

### 3.1. Participant Characteristics

The sample consisted of 137 students (mean age = 26.02, SD = 10.208). The majority of participants were female (N = 106, 77.4%), with the remainder being male (N = 31, 22.6%). The sample was divided between students of Medicine (N = 85, 62%) and Nursing (N = 52, 38%). Descriptive statistics for key continuous variables are presented in Table 1. Categorical distributions for BMI, anxiety, and depression showed that most participants were of normal weight (55.5%), had normal levels of anxiety (58.4%), and normal levels of depression (86.1%).

### 3.2. Correlations Between Body Image, BMI, and Psychological Distress

Spearman’s correlation analysis revealed significant associations between subjective body image concerns and symptoms of anxiety and depression. Specifically, higher scores on the HADS anxiety subscale (HADS-A) were positively correlated with Eating Concern (ρ = 0.334, *p* < 0.001), Shape Concern (ρ = 0.320, *p* < 0.001), and Weight Concern (ρ = 0.318, *p* < 0.001).

**Figure 1:**
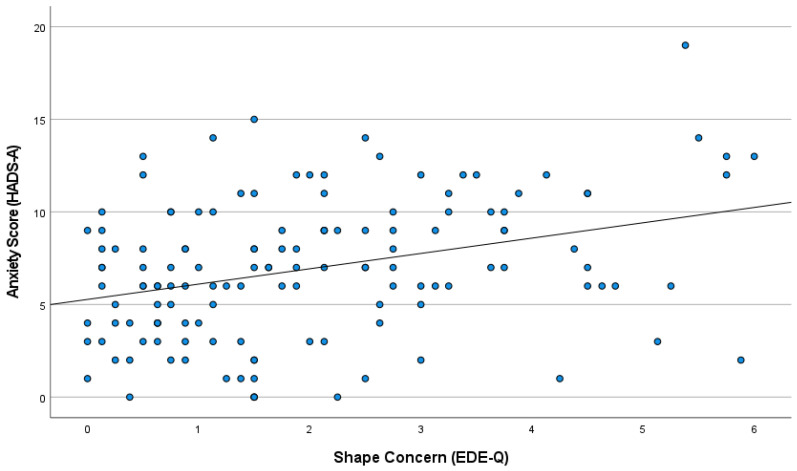
Scatter plot illustrating the positive correlation between HADS-Anxiety scores and EDE-Q Shape Concern scores. Each point represents a participant. The regression line indicates the overall trend (N = 137).

As shown in Figure 1, there was a clear positive association between anxiety symptoms (HADS-A) and Shape Concern scores (EDE-Q), with higher levels of subjective body image concern corresponding to greater psychological distress. Despite the moderate sample, the correlation between subjective body image concerns and anxiety (ρ = 0.032, *p* < 0.001) remained strong and statistically significant, consistent with patterns observed in larger studies.

Similarly, higher scores on the HADS depression subscale (HADS-D) were positively correlated with Eating Concern (ρ = 0.267, *p* = 0.002), Weight Concern (ρ = 0.247, *p* = 0.004), and Shape Concern (ρ = 0.199, *p* = 0.020).

**Figure 2:**
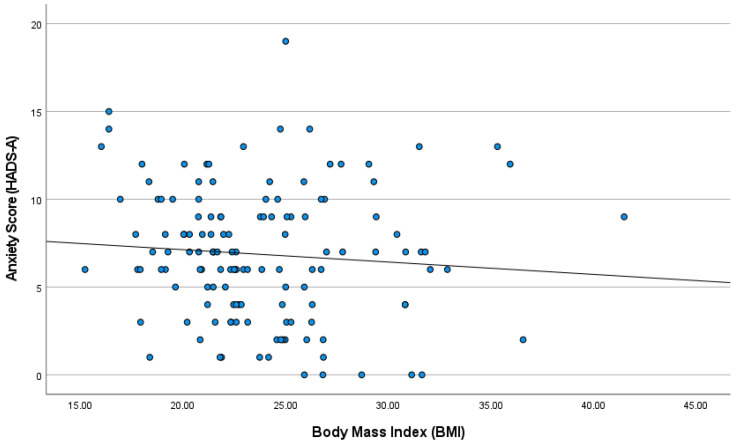
Scatter plot showing the absence of correlation between HADS-Anxiety scores and BMI. Each point represents a participant; the regression line is nearly horizontal (N = 137).

In striking contrast, BMI exhibited no statistically significant correlation with either HADS-A scores (ρ = −0.127, *p* = 0.138) or HADS-D scores (ρ = 0.065, *p* = 0.448). In contrast, as illustrated in Figure 2, BMI showed no clear relationship with anxiety symptoms, with data points dispersed and the regression line close to flat, visually confirming the lack of a statistically significant association. The absence of a statistically significant association between BMI and psychological distress further reinforces the primacy of subjective perceptions, a result that holds despite the sample size.

The full correlation results are presented in Table 2.

### 3.3. Descriptive Statistics for Medicine and Nursing Students

As it was already presented in Table 1, there were 95 students enrolled in the Medicine programme and 42 in the Nurse programme. Descriptives for the Medicine programme are presented in Table 3 and for Nurse in Table 4.

### 3.4. Group Comparisons Between Medicine and Nursing Students

**Figure 3:**
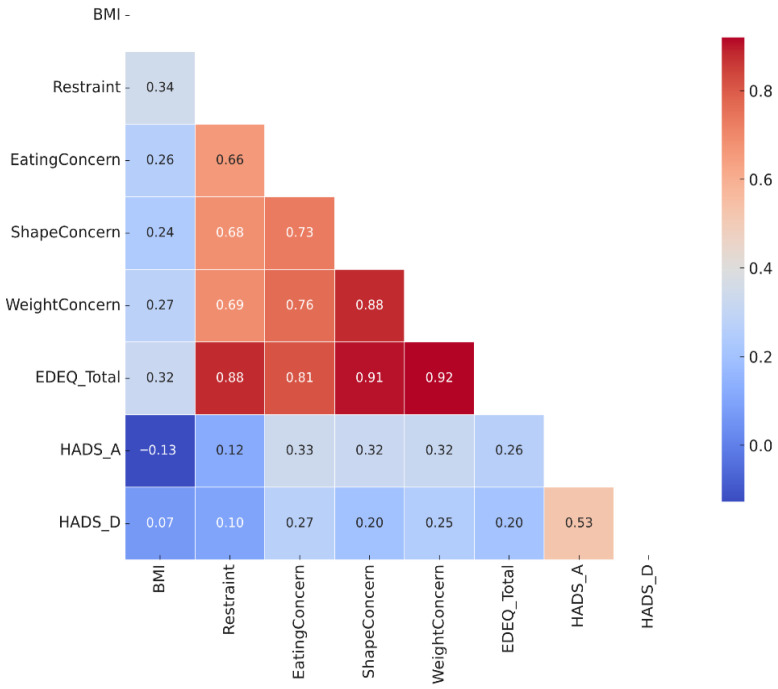
Heatmap of Spearman’s correlation coefficients between BMI, HADS subscales, and EDE-Q subscales. Statistically significant correlations are highlighted (N = 137).

For a comprehensive overview of the relationships among the main variables, Figure 3 presents a heatmap of Spearman’s correlation coefficients.

Mann–Whitney U tests were employed to compare variables between students of Medicine (N = 85) and Nursing (N = 52). A statistically significant difference in BMI was found between the two groups, with Nursing students having a higher median BMI (Median = 24.98) compared to Medicine students (Median = 22.36), U = 1354.00, *p* = 0.004.

**Figure 4:**
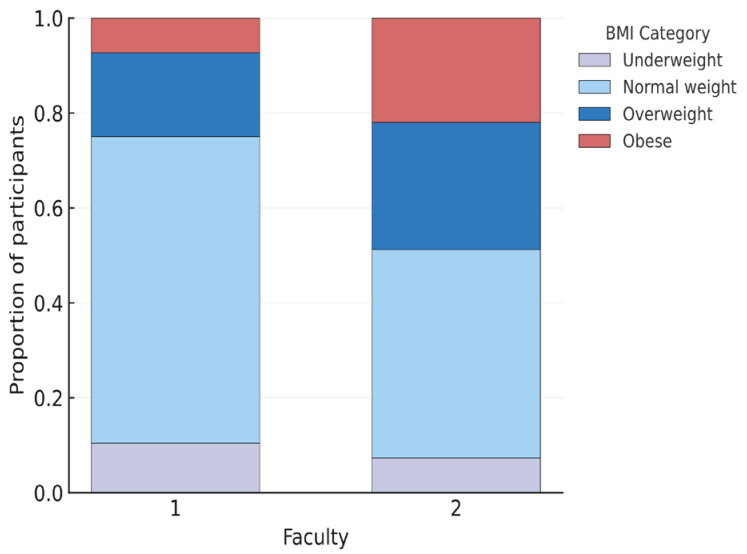
Distribution of body-mass index (BMI) categories by faculty (1, Medicine; 2, Nursing).

The distribution of BMI categories by faculty is shown in Figure 4, highlighting a higher proportion of overweight and obese individuals among nursing students.

**Figure 5:**
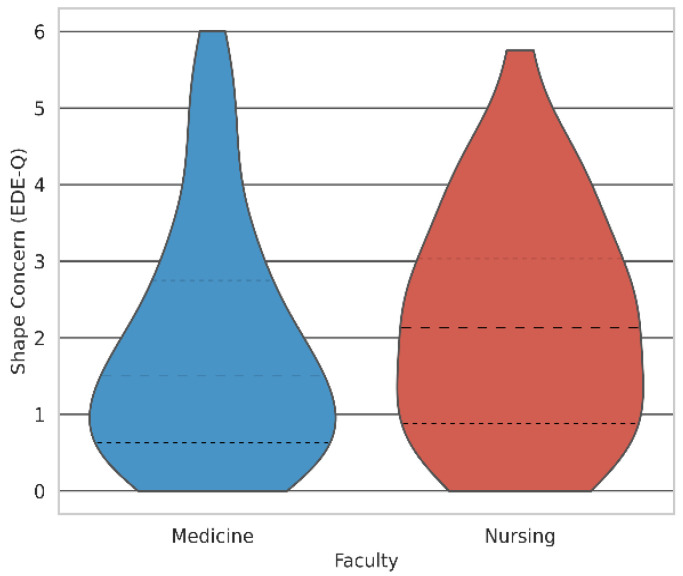
Violin plot of Shape Concern (EDE-Q) scores by faculty, visualising distribution shape, outliers, and median (N = 137).

Figure 5 displays the distribution of Shape Concern scores (EDE-Q) by faculty, revealing asymmetry and greater variability among nursing students.

**Figure 6:**
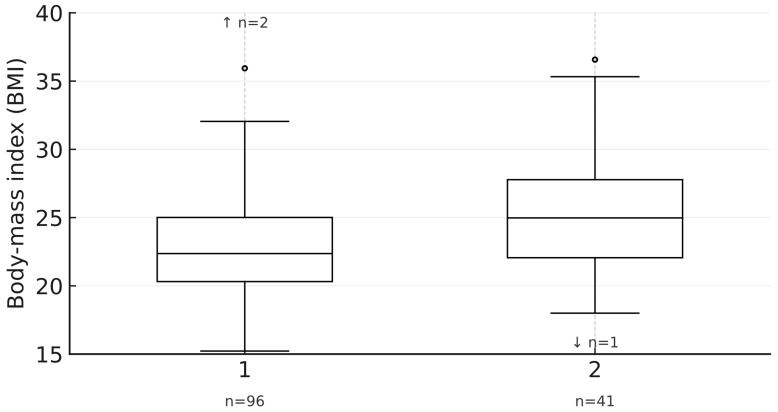
Box-and-whisker plots of body-mass index (BMI) by faculty (1 = Medicine; 2 = Nursing).

Figure 6 visually demonstrates significantly higher BMI in nursing students compared to medical students.

**Figure 7:**
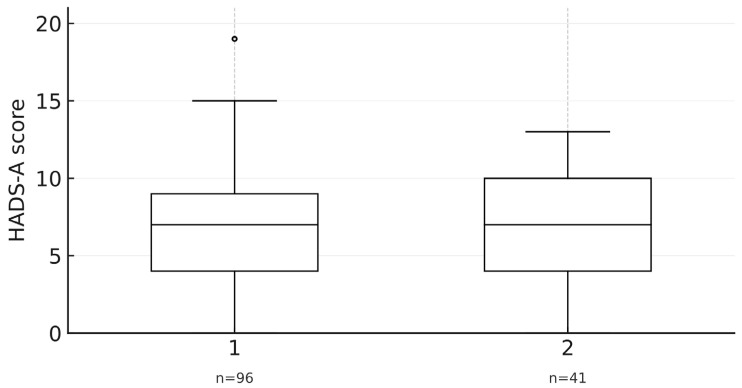
Box plot of HADS-Anxiety scores by faculty, illustrating similar distributions for Medicine and Nursing students (N = 137).

However, no statistically significant differences were found between the two groups for any of the psychological variables, including HADS-A and HADS-D scores, or for any of the EDE-Q subscales (all *p* > 0.05). However, Figure 7 shows that anxiety scores (HADS-A) were very similar between the two groups, with overlapping distributions and medians.

These results are detailed in Table 5.

## 4. Discussion

This study investigated the relationships between body image, BMI, and symptoms of anxiety and depression in a sample of Romanian health sciences students. The principal finding is the clear dissociation between the impact of subjective body perception and that of objective body mass on mental health. Our results demonstrate that subjective concerns regarding shape, weight, and eating are significantly and positively correlated with higher levels of anxiety and depression. Conversely, BMI, an objective measure of weight status, showed no significant association with emotional distress. This finding strongly supports the study’s central hypothesis and suggests that, for this cohort of future health professionals, the psychological and emotional experience of one’s own body is a more salient correlate of mental well-being than actual weight status. Notably, the robust associations observed in this moderate sample suggest that subjective body image dissatisfaction is a key driver of anxiety and depression among health sciences students, irrespective of objective weight status.

### 4.1. The Primacy of Perception in the Context of Global Literature

This central result aligns with a growing body of international literature that acknowledges the limitations of BMI as a predictor of mental health and underscores the importance of body dissatisfaction as a risk factor for negative psychological outcomes [33,34,35]. Importantly, in Romania, Pop (2016) reported that body image dissatisfaction correlated more strongly with self-esteem (r = −0.36, *p* < 0.001) than BMI, reinforcing the regional applicability of our results [36]. Furthermore, similar findings have been documented in China, where body dissatisfaction predicted anxiety and depression across genders [37,38,39]. The consistency of these results—across genders, cultures, and BMI statuses—supports the view that subjective body perception is a more potent driver of psychological distress than objective body measures.

Furthermore, the finding of no significant differences in psychological distress between medical and nursing students, despite a significant difference in their mean BMI, serves as a powerful demonstration of this dissociation. This reinforces the argument that it is not the physical metric itself, but the subjective experience thereof, that is the primary driver of the observed mental health outcomes. It suggests that common factors related to the medical academic environment (e.g., stress, perfectionism) or socio-cultural pressures may exert a more uniform impact on the students’ psyche than do their physical differences [25,40,41,42].

### 4.2. Regional Novelty: Body Image in the Eastern European Context

This study makes a unique contribution by focusing on an under-researched population in a distinct regional context. As summarised in Table 6, research from Eastern Europe is beginning to form a complex picture of body image concerns.

Our results fit within this regional landscape but add a crucial nuance. The amplified importance of subjective perception in our Romanian sample may reflect the ongoing socio-cultural transition in the region. As Western, often unrealistic, beauty ideals permeate through media and globalisation, they may create a significant and distressing discrepancy between the internalised ideal and physical reality [46,47]. This discrepancy is a potent source of psychological distress, which explains why subjective perception becomes a stronger correlate of anxiety and depression than objective BMI [14,19,48]. Moreover, our findings complement the study by Motorga et al. (2025), which identified familial pressure as a key risk factor for eating disorders in Romanian medical students [23]. Our study suggests that this external pressure manifests as an internal, subjective dissatisfaction, which is in turn tightly linked to symptoms of anxiety and depression.

### 4.3. Implications for University Mental Health in Romania

The strong association between body image dissatisfaction and mental health symptoms found in this cohort underscores the need for practical clinical action within the university setting. Therefore, university health services should consider screening for body image dissatisfaction using validated instruments such as the EDE-Q, not merely monitoring BMI, to identify students at risk. Interventions should target the cognitive (e.g., cognitive restructuring of negative body-related thoughts) and affective (e.g., developing self-compassion) components of body image.

### 4.4. Strengths and Limitations

The study has several strengths, including the use of validated instruments, a focus on a key and vulnerable demographic, and its regional novelty in providing data from an under-researched Eastern European context. However, certain limitations must also be acknowledged. The cross-sectional design precludes the establishment of causal relationships. The reliance on self-reported data for BMI calculation introduces the possibility of inaccuracies. The recruitment method may have introduced a self-selection bias, and the sample being drawn from a single institution limits the generalisability of the results. Furthermore, the study did not account for other potential confounding factors, such as academic stress, social support, or pre-existing mental health conditions. Another significant limitation of this study is the high prevalence of female participants (77.4%), which is characteristic of health sciences programmes in the region but affects the generalizability of findings to the wider student population, especially male students. Future research should aim for larger, more balanced samples. Also, another limitation is the scope of the instruments used. While subjective body image was assessed, the study did not include measures of external factors, such as social comparison or media-related pressure on body image. These socio-cultural influences are critical contextual factors that contribute to body dissatisfaction, and their omission limits the depth of interpretation regarding the formation of body dissatisfaction. Future longitudinal studies should integrate measures of these external pressures.

Although the cross-sectional design and moderate sample size may limit generalizability, the consistency of the observed effects and use of validated instruments support the reliability of the study’s main conclusions.

### 4.5. Future Research Directions and Clinical Implications

Based on these findings and the high prevalence of associated distress observed in our cohort, longitudinal studies are recommended to track the evolution of the relationship between these variables over time. Qualitative research could also provide a deeper understanding of the lived experience of body image concerns in this specific cultural and academic context.

Clinically, the development and implementation of scalable, low-barrier interventions is essential to address the strong association between body image dissatisfaction and mental health symptoms. Digital Mental Health Interventions (DMHIs) have demonstrated significant efficacy for both depression and anxiety in university students [49]. Since body image-related anxiety is often rooted in fears of social evaluation, automated DMHIs—which have been noted for their effectiveness in reducing anxiety—offer a promising avenue by potentially removing the fear of judgment from a therapist. Therefore, future research should test the efficacy of culturally adapted, automated DMHIs specifically targeting body dissatisfaction in the Romanian student population.

## 5. Conclusions

This study demonstrates strong associations that, among Romanian health sciences students, subjective perception and the emotional response to one’s own body are far more strongly correlated with psychological distress than objective weight status. Intense preoccupations with weight, shape, and eating were consistently associated with elevated symptoms of anxiety and depression, indicating that negative body image constitutes a significant psychological factor in this population. The dissociation between BMI and emotional distress underscores the limited power of objective metrics in predicting mental health outcomes. These findings highlight the urgent need for mental health initiatives within academic institutions to address body image concerns as a central element of student psychological health, promoting strategies that cultivate healthier body perceptions and emotional resilience. In summary, body dissatisfaction is strongly associated with increased symptoms of anxiety and depression in this population, suggesting that subjective perception is a more relevant mental health predictor than BMI. We advocate for future longitudinal studies to confirm the directionality and causality of these relationships.

## Figures and Tables

**Table 1:** Participant Demographics and Descriptive Statistics for Key Variables.

Variable	N	Mean (SD) or N (%)	Interval
Age (years)	137	26.02 (10.21)	18–53
Gender	137		
Female		106 (77.4%)	
Male		31 (22.6%)	
Study programme	137		
Medicine		95 (69.0%)	
Nurse		42 (31.0%)	
BMI (kg/m^2^)	137	24.10 (7.19)	15.22–41.5
Score HADS-Anxiety	137	6.39 (3.76)	0–19
Score HADS-Depression	137	4.17 (2.95)	0–13
EDE-Q Form Concern	137	1.84 (1.67)	0–6
EDE-Q Weight Concern	137	1.63 (1.69)	0–6
EDE-Q Alimentation Concern	137	0.81 (1.18)	0–5.6
BMI Categories	137		
Underweight (<18.5)		13 (9.5%)	
Normal Weight (18.5–24.9)		76 (55.5%)	
Overweight (25.0–29.9)		32 (23.4%)	
Obese (≥30.0)		16 (11.7%)	
Anxiety Categories (HADS-A)	137		
Normal (0–7)		80 (58.4%)	
Borderline (8–10)		32 (23.4%)	
Abnormal (≥11)		25 (18.2%)	
Depression categories (HADS-D)	137		
Normal (0–7)		118 (86.1%)	
Borderline (8–10)		13 (9.5%)	
Abnormal (≥11)		6 (4.4%)	18–53

**Table 2:** Correlation analyses between burnout and various variables.

Variable	BMI	EDE-Q Restraint	EDE-Q Alimentation Concern	EDE-Q Form Concern	EDE-Q Weight Concern
Score HADS-A					
ρ	−0.127	0.121	0.334	0.320	0.318
*p*	0.138	0.160	<0.001	<0.001	<0.001
Score HADS-D					
ρ	0.065	0.099	0.267	0.199	0.247
*p*	0.448	0.248	0.002	0.020	0.004

Note: Correlations significant at the *p* < 0.05 level are in italic.

**Table 3:** Descriptive statistics for Medicine students.

Variable	N	Mean (SD) or N (%)	Interval
Age (years)	95	21.58 (5.13)	18–38
Gender	95		
Female		67 (70.5%)	
Male		28 (29.5%)	
BMI (kg/m^2^)	95	23.02 (4.27)	15.22–41.5
Score HADS-Anxiety	95	6.97 (3.64)	0–19
Score HADS-Depression	95	4.08 (2.80)	0–13
EDE-Q Form Concern	95	1.87 (1.52)	0–6
EDE-Q Weight Concern	95	1.61 (1.48)	0–6
EDE-Q Alimentation Concern	95	0.83 (1.17)	0–5.6
BMI Categories	95		
Underweight (<18.5)		10 (10.5%)	
Normal Weight (18.5–24.9)		59 (62.1%)	
Overweight (25.0–29.9)		20 (21.1%)	
Obese (≥30.0)		6 (6.3%)	
Anxiety Categories (HADS-A)	95		
Normal (0–7)		55 (57.9%)	
Borderline (8–10)		26 (27.4%)	
Abnormal (≥11)		14 (14.7%)	
Depression categories (HADS-D)	95		
Normal (0–7)		82 (86.3%)	
Borderline (8–10)		10 (10.5%)	
Abnormal (≥11)		3 (3.2%)	

**Table 4:** Descriptive statistics for Nurse students.

Variable	N	Mean (SD) or N (%)	Interval
Age (years)	42	36.32 (11.64)	19–53
Gender	42		
Female		39 (92.86%)	
Male		3 (7.14%)	
BMI (kg/m^2^)	42	25.73 (4.54)	18.00–36.57
Score HADS-Anxiety	42	6.60 (4.01)	0–13
Score HADS-Depression	42	4.08 (3.09)	0–12
EDE-Q Form Concern	42	2.17 (1.46)	0–5.75
EDE-Q Weight Concern	42	1.71 (1.45)	0–5.8
EDE-Q Alimentation Concern	42	0.92 (1.18)	0–4.2
BMI Categories	42		
Underweight (<18.5)		3 (7.1%)	
Normal Weight (18.5–24.9)		17 (40.5%)	
Overweight (25.0–29.9)		12 (28.6%)	
Obese (≥30.0)		10 (23.8%)	
Anxiety Categories (HADS-A)	42		
Normal (0–7)		25 (59.5%)	
Borderline (8–10)		6 (14.3%)	
Abnormal (≥11)		11 (26.2%)	
Depression categories (HADS-D)	42		
Normal (0–7)		36 (85.7%)	
Borderline (8–10)		3 (7.1%)	
Abnormal (≥11)		3 (7.1%)	

**Table 5:** Mann–Whitney U Test Comparisons of Key Variables between Medicine and Nursing Students.

Variable	Medicine Mean (IQR)	Nurse Mean (IQR)	U-Statistic	*p*
BMI (kg/m^2^)	22.36 (4.72)	24.98 (6.73)	1354.0	0.004
Score HADS-A	7 (5)	7 (7)	1942.0	0.902
Score HADS-D	4 (4)	4 (5)	1960.5	0.972
EDE-Q Form Concern	1.50 (2.12)	2.13 (2.50)	1665.5	0.155
EDE-Q Weight Concern	1.20 (2.20)	1.60 (2.00)	1819.0	0.483
EDE-Q Alimentation Concern	0.40 (1.40)	0.40 (1.60)	1883.0	0.685

Note: Differences significant at the *p* < 0.05 level are in italic. IQR = Interquartile Range.

**Table 6:** Summary of Recent Findings on Body Image Dissatisfaction and Correlates among Students in Eastern Europe.

Country/Region	Sample	Key Findings	Source
Romania	Medical students	High risk of eating disorders (37.1%), higher than in the general population. Family pressure is a stronger predictor than media pressure.	[23]
Poland	University students	High body dissatisfaction; women are more willing to diet. Only 1 in 5 students is fully satisfied with their appearance. Greater tolerance for overweight than for underweight.	[18,19]
Czech Republic	Adolescents	Females with normal BMI want to lose weight. Women are more critical of their bodies. Criticism from others (parents, peers) amplifies dissatisfaction.	[20]
Slovakia	Adolescents	Over 35% of girls perceive themselves as “too fat.” Weight-control behaviours are common but do not necessarily align with actual obesity.	[20]
Lithuania	Adolescents	Higher BMI and weight overestimation are associated with greater body dissatisfaction and lower self-esteem. Body dissatisfaction does not promote healthy behaviours.	[43]
Hungary	University students	Linear relationship between body dissatisfaction and BMI in both men and women. Low self-determination is associated with greater dissatisfaction.	[44]
Moscow/Tiraspol	University students	Similar levels of dissatisfaction in both sexes (69% men, 67% women), but for different reasons: men due to underweight, women due to overweight.	[21]
Croatia	High school students	Lack of an association between low self-esteem and self-evaluation of appearance, possibly due to traditional values.	[45]

## Data Availability

The raw dataset generated during the current study is available in the Zenodo repository at https://doi.org/10.5281/zenodo.17541525. Access to the dataset is restricted to protect participant privacy. Data can be made available to researchers upon reasonable request submitted through the Zenodo access request system.

## Supplementary Materials

The following supporting information can be downloaded at https://www.mdpi.com/article/10.3390/healthcare13233118/s1, STROBE Statement—checklist of items that should be included in reports of observational studies.
